# Linking proactive personality to successful aging at work: a moderated mediation model

**DOI:** 10.3389/fpsyg.2025.1616092

**Published:** 2025-09-03

**Authors:** Lijun Zhuo, Qian Wu, Yating Cong, Xinyu Bao

**Affiliations:** ^1^School of Medicine and Health Management, Tongji Medical College, Huazhong University of Science and Technology, Wuhan, China; ^2^Shenzhen Health Development Research and Data Management Center, Shenzhen, China; ^3^Affiliated Hospital of Shandong University of Traditional Chinese Medicine, Jinan, China; ^4^School of Public Health and Nursing, Hubei University of Science and Technology, Xianning, China

**Keywords:** proactive personality, successful aging at work, job crafting, organization support, lifespan development theory

## Abstract

Using lifespan development theory, we explored how proactive personality influences successful aging at work among older physicians in public hospitals. Job crafting as a mediator and perceived organizational support as a moderator in this relationship. A moderated mediation model was developed and tested on a sample of 472 older physicians in 13 public hospitals in China. Hierarchical regression analysis was employed to test the study hypotheses. Regression analysis revealed a positive association between proactive personality and successful aging at work, with job crafting serving as a positive mediator in this relationship. Perceived organizational support moderated the indirect effect of proactive personality on successful aging at work via job crafting. Older physicians perceive higher level of organization support, the indirect effect was stronger. Our study makes important contributions to the extant literature by indicating whether, how, and when proactive personality has an effect on successful aging at work of older physicians. By the personality traits of older physicians, organizations should offer targeted guidance and training to help them adjust their roles and facilitate their adaptation to changes in the workplace.

## 1 Introduction

Global population are aging as a result of persistently low or declining fertility rates, the aging of the baby boomer generation, and rising life expectancy. Between 2015 and 2050, the globe population aged over 60 is expected to almost double, increasing from 12% to 22% (World Health Organization, 2015). The normal human aging categorized into two types: “usual aging,” where extrinsic factors exacerbate aging effects, and “successful aging,” where they have neutral or positive effects (Rowe and Kahn, 1987). Successful aging involves three main components: minimizing the likelihood of disease and disability, preserving high levels of physical and cognitive function, and remaining actively involved in social and productive activities (Payne et al., 1997).

The slowing growth of the workforce is beginning to lag behind overall employment growth, compounded by an aging population, resulting in persistent labor shortages (Kooij et al., 2020). Consequently, older workers are required to stay in the workforce for as long as possible and motivated to continue working to address this challenge (Kooij et al., 2020). However, older workers are often seen as less motivated, healthy, and adaptable, with a lower willingness to embrace change, and considered more susceptible to work-family imbalances (Ng and Feldman, 2012). As a result, scholars and practitioners are increasingly focused on successful aging in the workplace (Zacher and Rudolph, 2017). The concept of Successful Aging at Work (SAW) was first introduced by Abraham and Hansson, suggesting that aging individuals can maintain job performance and employment competence (Abraham and Hansson, 1995). There are two perspectives define SAW: the maintenance and developmental views. The maintenance view refers to older employees' ability to sustain their health, motivation, and work capacity both in the present and going forward (Kooij, 2015). The developmental view posits that not only maintaining current functioning but also achieving growth, such as advancement in specific areas (Zacher, 2015a).

However, very few studies have concentrated on SAW. Recent studies have begun to explore various definitions and conceptualizations of SAW, as well as its antecedents (Zacher, 2015a). Previous studies suggest that different levels of antecedents, including environmental factors (e.g., cultural values, legislation), organizational factors (e.g., organizational support, human resource management practices), job factors (e.g., job design, person-job fit), and personal factors (e.g., individual traits, psychology, behavior) may influence SAW (Beier et al., 2018; Cheung and Wu, 2012; Kooij et al., 2020; Zacher et al., 2018). The interaction of these factors remain unclear, and empirical verification is lacking.

Personal resources are particularly crucial for older workers as they navigate age-related challenges and losses (Kooij et al., 2020). Previous research suggests that proactive personality, which influences work outcomes differently across age groups, serves as a vital personal resource for SAW (Zacher, 2015b). Proactive personality is an individual difference factor that drives positive situational changes by fostering proactive behaviors, unconstrained by environmental factors (Bateman and Crant, 1993; Wang et al., 2017). The major domains related to proactive personality comprise corporate social responsibility, entrepreneurship, career development and performance, job crafting and work engagement, leadership and innovation, socialization and information seeking. The major themes in career development include career satisfaction, career success, and turnover intention (Din et al., 2023; Kim and Oh, 2024). Whether proactive staffing has a positive effect on older employees achieving career success, (i.e.,) successful aging at work, remains to be explored.

Physicians, as knowledge-based employees, gain clinical knowledge as their years of experience increase, and have a high need for job fulfillment and career development. Older physicians who extend their careers make vital contributions to healthcare delivery and play an essential role in the education and mentoring of younger colleagues. However, many physicians begin planning their retirement due to deteriorating health, cognitive impairment, declining adaptability, lower job satisfaction, and a growing desire for personal or leisure time (Joyce et al., 2015; Wijeratne et al., 2017). The decision to retire involves a complex factors related to aging, including physical and cognitive changes that may impede optimal practice (Wijeratne et al., 2017). Therefore, how to better enable older physicians to realize their value and SAW later in their careers should be certainly-concerned. Lifespan development theory emphasizes that aging employees achieve their goals by maximizing gains and minimizing losses. A proactive personality may lead older workers to exhibit more proactive behaviors, enabling them to make lifestyle choices that slow the decline in competence and motivation, potentially contributing to successful aging (Zacher and Kooij, 2016). Although researchers have proposed that proactive personality may evolve with age and facilitate SAW, the underlying mechanisms lack empirical research (Zacher, 2015b).

Taken together, our study aims to explore how proactive personality impacts SAW among physicians, and makes contributions to the literature in two aspects. First, we present proactive personality as a novel antecedent of SAW, responding to the previous studies found that proactive personality was positively related to initiative job search or career success (Kanfer et al., 2001; Kooij et al., 2020; Seibert et al., 2001). Proactive older workers are more inclined to adjust job content and the work environment to align with organizational needs and achieve their own career goals. Then, we introduce job crafting as a mediator between proactive personality and SAW to explore how proactive personality influences the SAW of aging physicians in hospitals, which enrich the lifespan development theory (Zacher and Kooij, 2016). Second, we demonstrate that organization support, as an external resource, can compensate for the physical and work competence losses of older workers. Our study introduces POS as an important moderator to strengthen the positive relationship between proactive personality and job crafting among physicians. By examining this moderating effect, we contribute to a deeper understanding of when proactive personalities are more inclined to be expressed and broaden the existing literature on organization support as an antecedent to SAW (Cheung and Wu, 2013).

## 2 Theoretical hypotheses development

### 2.1 Proactive personality and successful aging at work

Lifespan development theory, proposed by Baltes, suggests that individual development is not limited to childhood and adolescence but continues through middle and old age, encompassing the entire life course from gestation to death (Baltes and Baltes, 1990). It presents development as a continuum across the lifespan. From this perspective, the Selective Optimization with Compensation (SOC) model was introduced, which posits that aging is not solely a process of resource loss (e.g., disease and cognitive decline), but also involves the acquisition of new resources (e.g., re-education and training), and proactive personality plays an important role (Baltes et al., 1999; Marsiske et al., 1995). Successful aging, therefore, is the process of achieving a dynamic balance between gains and losses (Baltes et al., 1999; Marsiske et al., 1995).

SAW is influenced by a combination of interacting factors, including individual, organizational, and other contextual factors, each contributing in unique ways (Kooij et al., 2020). Lifespan development theory effectively explains how older employees can adapt positively to age-related changes. Even late in their careers, older employees can still realize their value and contribute to the organization by proactively leveraging their strengths and weaknesses, maintaining motivation, and sustaining performance. Proactive employees are more inclined to actively pursue job opportunities (Kanfer et al., 2001), and are positively related to innovation and career success (Seibert et al., 2001). A survey of chief physicians in German hospitals indicated that proactive personality had a positive effect on innovative work behavior (Kriegl et al., 2024). Junior physicians with proactive personality could gain more support from senior physicians by establishing a network, providing clinical training and funding their studies with grants (Buddeberg-Fischer et al., 2009). Thus, we proposed that physicians with proactive personalities are adept at identifying and seizing external opportunities, proactively creating favorable conditions to achieve their goals rather than passively reacting to their environment.

*H1*. Proactive personality is positively related to SAW.

### 2.2 The mediating role of job crafting

The idea of job crafting was first introduced by Kulik, Oldham, and Hackman, who suggested that employees have the autonomy to redesign their jobs and that by actively participating in shaping their roles, they can better align their individual needs with their functions within the organization (Kulik et al., 1987). The idea of job crafting is expanded to include the actions individuals take to modify the scope of their tasks, relationships, and the meaning of work, and enables employees to proactively adjust their work in alignment with their preferences, abilities, and characteristics, rather than passively accepting the organization's management (Wrzesniewski and Dutton, 2001).

Job crafting is considered a bottom-up approach to work design, reflecting the proactive behavior of individual employees (Zhang and Parker, 2019). The antecedents of job crafting can be categorized into distal and proximal variables, with proactive motivation being a more proximal antecedent than job characteristics or leadership behavior (Zhang and Parker, 2019). A range of personality traits and beliefs act as individual factors that influence the likelihood of engaging in job crafting (Rudolph et al., 2017). Empirical evidence suggested that proactive personality could predict organizational behaviors (Bakker et al., 2012). Physicians with proactive personality could integrate personal and organizational resources to adjust their employability to fit the position need, for instance, through job crafting behavior (Bakker et al., 2012). Empirical evidence indicated that employees with proactive personalities are positively linked to all forms of job crafting. Such individuals often exhibit high levels of initiative, actively enhance their job resources, identify opportunities, overcome barriers, and take on additional job challenge until they achieve their goals (Bakker et al., 2012; Crant et al., 2016; Rudolph et al., 2017).

Researchers have increasingly focused on the role of job crafting among older employees. SAW of older physicians is closely associated with ongoing work practices, and that physicians could mitigate the stresses of aging by working fewer hours, increasing nonclinical activities, and recruiting new colleagues (Wijeratne et al., 2018). Adjusting their roles, such as by nurturing and mentoring younger colleagues was also a way to motivate to continue working (Shkop, 1982). Older employees can achieve a sense of job coherence through job crafting, which fosters improved health and sustained motivation to remain employed beyond retirement age (Lichtenthaler and Fischbach, 2016). Older employees engage in more relational and cognitive crafting to cope with declining physical abilities and to pass on intellectual skills, whereas younger employees are primarily motivated by personal career development needs (Van den Oetelaar, 2011). Previous research found that job crafting mediates the association between proactive personality and work engagement. Additionally, colleague ratings of proactive personality were positively linked to job performance, with job crafting and work engagement acting as intermediary factors (Bakker et al., 2012).

*H2*. Job crafting mediates the relationship between proactive personality and SAW.

### 2.3 The moderating role of perceived organizational support

Aging-related contextual resources are increasingly necessary for older workers, as age-related issues tend to be more prominent in the workplace (Zacher and Yang, 2016). Perceived Organizational Support (POS), an important contextual factor, reflects whether an organization provides a supportive environment and sufficient job resources when needed (Wang et al., 2017). Previous research indicated that more perceptions of organizational support for will lead healthcare workers to putting in more effort to facilitate knowledge sharing, improve performance and help the organization create a competitive advantage (Tian et al., 2018). A meta-analysis revealed a link between physician burnout and career disengagement. The study also found that burnout was more strongly associated with lower job satisfaction, particularly among older physicians working in emergency medicine and intensive care, compared to those experiencing increased job satisfaction (Hodkinson et al., 2022). A supportive organizational climate that fosters successful aging can help mitigate the adverse impacts of aging on aspects like job satisfaction, organizational commitment, and the motivation to remain in the workforce (Zacher and Yang, 2016). Supportive leadership and an autonomy-oriented culture, as types of organization support could enhance the positive relationship between proactive personality and job performance (Kim et al., 2014). Job crafting, aimed at aligning with job needs, is hypothesized to be positively influenced by proactive personality. POS enhances the impact of proactive personality on job crafting among older physicians. Furthermore, previous studies indicate that job crafting fully mediates the association between proactive personality and job performance, while organizational embeddedness moderates the link between proactive personality and job crafting (Yu et al., 2022). Therefore, we proposed that proactive older physicians may be more inclined to seize opportunities and engage in proactive career behaviors to shape their environment, and perform well in higher level of POS, thereby achieving SAW.

*H3*. POS moderates the relationship between proactive personality and job crafting. When POS is higher rather than lower, the connection between proactive personality and job crafting is stronger.
*H4*. POS exposure moderates the indirect relationship between proactive personality and SAW through job crafting. When POS is higher rather than lower, the indirect effect of proactive personality on SAW via job crafting is stronger.

**Figure 1:**
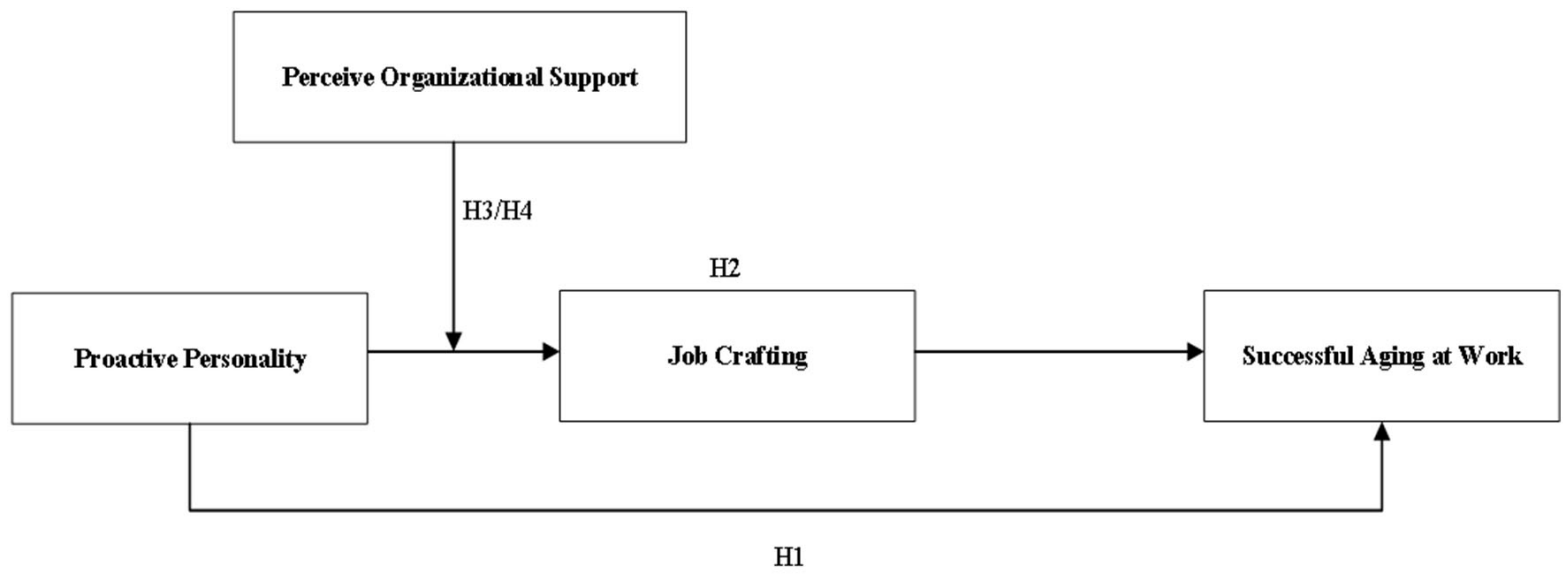
The hypothesis model.

Drawing from the preceding discourse and hypotheses, we drew the research model (see Figure 1).

## 3 Materials and methods

### 3.1 Participants and procedures

In the context of workplace aging, previous research commonly defines older employees as those who have not yet reached the statutory retirement age, with age 50 frequently used as the cutoff point (Thrasher et al., 2020). Although broader age ranges are applied (Robson et al., 2006), the extended training period required for medical professionals means that physicians typically enter the workforce later and possess greater accumulated expertise (Hsieh and Tang, 2019). As a result, their perception of aging may be delayed. Based on this understanding, we defined older physicians as those aged 50 and above. Data for this study were collected from 13 public hospitals located in Hubei, Shandong, and Jiangsu provinces in China. The survey was distributed with support from administrative staff at each hospital. Only physicians aged 50 or older were invited to participate, and no compensation was offered. To guarantee that participants were fully informed and their participation voluntary, the questionnaire's introduction outlined the study's objective, data collection scope, privacy risks, and mitigation measures the potential risks to privacy. The study was conducted in a fully anonymous format, and the data were exclusively utilized for academic research and not disclosed to the organizations from which the participants originated. Participants were informed of their right to withdraw, with acceptance of the survey considered as informed consent.

Inclusion criteria for participation were as follows: (a) the respondent was a regular employee, having been employed as a physician in the hospital for a period exceeding one year; (b) the respondents were aged 50 years and above and were employed in a clinical department; (c) the respondents were able to complete the entire questionnaire independently. To ensure data quality, we verified that all questionnaires were fully completed and excluded any responses that were internally inconsistent or showed signs of uniform or patterned answering.

In total, 585 questionnaires were distributed, of which 511 were successfully collected, and 472 were deemed valid, resulting in an effective response rate of 92.37%. In the final sample, 298 (63.14%) were male. Ages ranged from 50–63 years, 71.40% were aged 50–55 years. The average work tenure was 30.10 years (*SD* = 4.08), 48.52% of respondents had obtained a bachelor's degree.

### 3.2 Measures

The variables employed in this study were carefully derived from well-established and widely recognized scales. Items initially in English were back-translated into Chinese (Brislin, 1980).

*Proactive personality*. The six items version of proactive personality originally developed by Bateman and Crant (1993) were used in our research, which show strong correlations with the 17-items scale (Claes et al., 2005) and have been used in previous studies (Li et al., 2010; Parker, 1998). All items ranged from 1 = “strongly disagree” to 7 = “strongly agree”. A sample item is “I am always looking for better ways to do things.” The scale's Cronbach's alpha was 0.924.

*Perceived organizational support*. The POS was assessed using a six-item scale developed by Eisenberger et al. (2001). Prior studies have validated the reliability and validity of this scale (Li et al., 2023). All items ranged from 1 = “strongly disagree” to 7 = “strongly agree.” An example item is “The organization is willing to help me if I need a special favor.” The scale's Cronbach's alpha was 0.945.

*Job crafting*. Job crafting was measured with 4-items designed by Leana et al. (2009) and verified in China by Zhan et al. (2024). All items ranged from 1 = “never” to 7 = “quite frequently”. A sample item is “I change the way I work to make it easier for myself.” The scale's Cronbach's alpha was 0.963.

*Successful Aging at Work*. SAW was measured by the successful aging in the work place scale developed by Robson et al. (2006), which entails five dimensions. The first, named adaptability and health (e.g., “I search for opportunities to do my job better”), includes 18-items. The second, named positive relationships (e.g., “I have many friends at work”), includes seven-items. The third, named occupational growth (e.g., “My career is still growing”), includes eight-items. The fourth, named personal security (e.g., “I am not overwhelmed by my workload”), includes seven-items. The fifth, named personal goals (e.g., “I am able to achieve the goals that I have defined for myself”), includes three-items. All items ranged from 1 = “strongly disagree” to 7 = “strongly agree”. The scale's Cronbach's alpha was 0.948.

*Controls*. Incorporating gender, age, education, and years of employment as control variables is supported by prior research, which has shown that these demographic factors may influence the behavior of older employees (Zhao et al., 2023). Consequently, they were used as control variables in this study.

## 4 Results

Table 1 provides a comprehensive overview of the descriptive statistics, including the means, standard deviations, and pairwise correlations for each variable included in the proposed model. Proactive personality demonstrated positive correlation with POS (0.439, *p* < 0.01), job crafting (0.84, *p* < 0.01) and SAW (0.81, *p* < 0.01). POS demonstrated positive correlation with job crafting (0.76, *p* < 0.01) and SAW (0.466, *p* < 0.01). Job crafting was positively related to SAW (0.76, *p* < 0.01).

**Table 1:** Descriptive statistics and correlations.

**Variables**	**1**	**2**	**3**	**4**	**5**	**6**	**7**	**8**
1 Gender	1.000							
2 Age	−0.053	1.000						
3 Education	−0.207^**^	0.062	1.000					
4 Working years	0.021	0.658^**^	−0.096^*^	1.000				
5 Proactive personality	−0.094^*^	−0.013	0.027	−0.080	1.000			
6 Perceived organizational support	−0.138^*^	0.002	0.067	−0.019	0.439^**^	1.000		
7 Job crafting	−0.139^**^	−0.002	0.058	−0.041	0.840^**^	0.760^**^	1.000	
8 Successful aging in the workplace	−0.080	−0.028	0.042	−0.056	0.810^**^	0.466^**^	0.760^**^	1.000
Mean	**—**	54.108	**—**	30.097	5.274	5.165	5.569	5.586
SD	**—**	2.955	**—**	4.076	1.217	1.322	1.244	0.732

*N* = 472; ^*^p < 0.05; ^**^p < 0.01; SD, Standard Deviation.

### 4.1 Testing hypothesis

Table 2 shows the outcomes of our hypothesis testing, which consolidates the key findings from the analysis. In Model 3, after incorporating the control variables, the regression coefficient for proactive personality in relation to SAW (*b* = 0.353, *p* < 0.001) was found to be significantly positive, thereby providing strong empirical support for our Hypothesis 1. Consistent with our expectations, in Model 1, proactive personality was positively associated with job crafting (*b* = 0.855, *p* < 0.001), while in Model 3, job crafting was positively associated with SAW (*b* = 0.159, *p* < 0.001), further substantiating the hypothesized relationships. Furthermore, consistent with Preacher and Hayes (2008), a bootstrapping analysis with 5,000 replications was conducted to assess the indirect effect using the SPSS PROCESS Model 4. The analysis revealed a significant indirect effect of proactive personality on SAW through job crafting [0.136, bootstrapped 95% *CI* = (0.080, 0.194), excluding 0]. Thus, Hypothesis 2 was supported.

**Table 2:** Regression results of indirect and conditional indirect effects.

**Predictor variable**	**Job crafting**				**Successful aging in the workplace**	
	**Model 1**		**Model 2**		**Model 3**	
	**Beta**	**SE**	**Beta**	**SE**	**Beta**	**SE**
Gender	−0.144^*^	0.066	−0.040^*^	0.039	0.022	0.041
Age	−0.011	0.014	−0.009	0.008	−0.010	0.009
Education	0.048	0.040	0.018	0.024	0.017	0.025
Working years	0.015	0.010	0.011	0.006	−0.005	0.006
Proactive personality	0.855^***^	0.026	0.680^***^	0.020	0.353^***^	0.029
Perceived organizational support (POS)			0.484^***^	0.018		
Proactive personality × POS			0.035^***^	−0.010		
Job crafting					0.159^***^	0.029
*F*	228.631^***^		586.328^***^		164.371^***^	
*R^2^*	0.710^***^		0.898^***^		0.680^***^	
**Indirect and conditional indirect effects**			**Effect**	**SE**	**95% Boot CI**	
Indirect effect analyses							
Indirect effect of job crafting			0.136	0.029	[0.080, 0.194]	
Conditional indirect effect analyses							
High perceived organizational support (+1 SD)			0.115	0.025	[0.068, 0.166]	
Low perceived organizational support (-1 SD)			0.101	0.021	[0.061, 0.143]	
Difference			0.015	0.007	[0.003, 0.029]	

*N* = 472; ^*^p < 0.05; ^***^p < 0.001; SD, Standard Deviation; SE, Standard Error; CI, Confidence Interval.

Furthermore, the interaction effect between proactive personality and POS was found to be positively associated with job crafting (*b* = 1.023, *p* < 0.01) in Model 2. To examine the interaction in detail, we conducted simple slope analyses (Aiken, 1991). Results shown that proactive personality was more strongly related to job crafting when physicians had higher POS (*b* = 0.726, *p* < 0.001; 1 *SD* above mean) than when they had lower support (*b* = 0.634, *p* < 0.001; 1 *SD* below mean).

**Figure 2:**
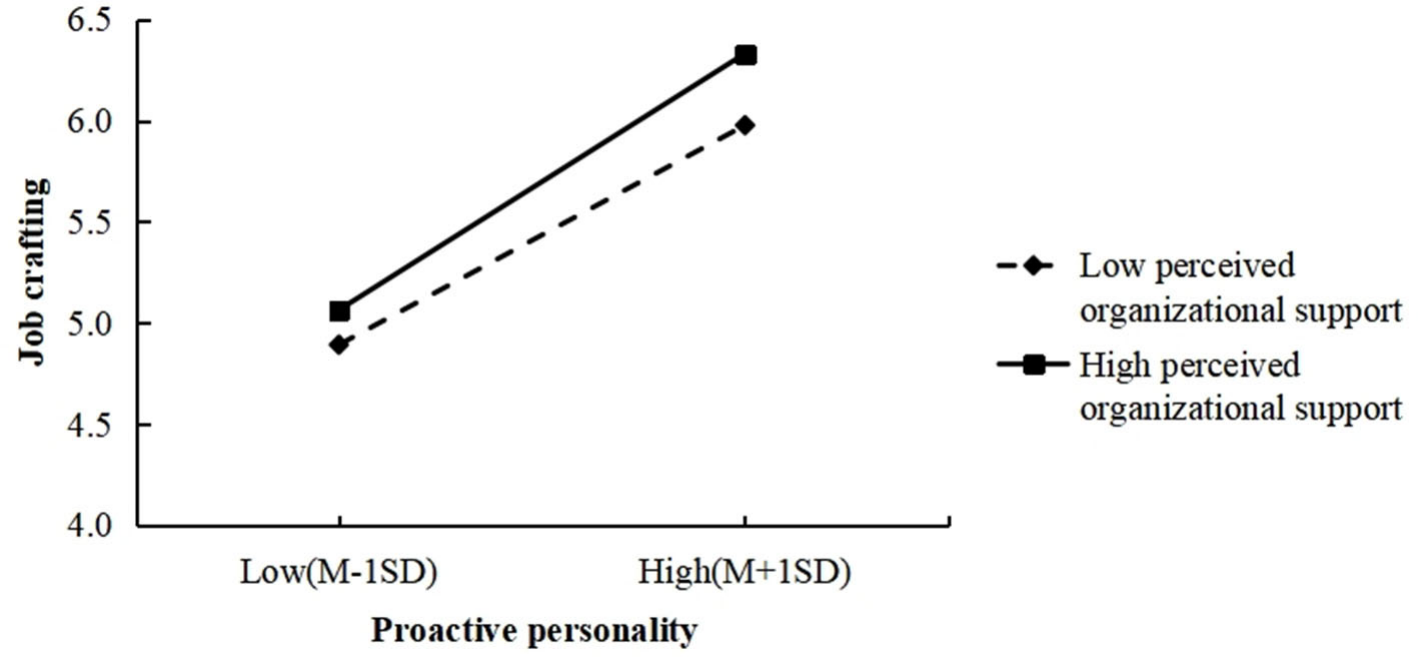
Moderating effect of perceived organizational support on the relationship between proactive personality and job crafting.

We further examined the moderated mediation hypothesis with the bootstrap methods. The lower part of Table 2 found that POS moderated the indirect effect of proactive personality on SAW through job crafting. The indirect effect was stronger when the value of POS was higher [estimate = 0.115, 95% *CI* (0.068, 0.166)] and lower [estimate =0.101, 95% *CI* (0.061, 0.143)]. The difference between the two effects was significant (difference = 0.015, 95% *CI* [0.003, 0.029]). Figure 2 showed that the moderating effect of POS on the relationship between proactive personality and job crafting.

## 5 Discussion

This study proposed a moderated mediation model, grounded in lifespan development theory, to investigate how physicians' proactive personalities influence their SAW (Baltes and Baltes, 1990). Consistent with our hypothesis, the results revealed a positive relationship between proactive personality and SAW, with job crafting mediating this relationship. Additionally, we hypothesized and confirmed that POS serves as a moderator in two key relationships: (1) between proactive personality and job crafting, and (2) the indirect effect of proactive personality on SAW.

### 5.1 Theoretical implication

Firstly, previous research has indicated that motivation for SAW is shaped by an interplay of both individual and situational factors (Zacher et al., 2018). Through empirical analysis, our study established that proactive personality as an individual factor positively correlate with SAW. This finding confirms the hypothesis of previous theoretical studies on proactive personality and SAW (Kooij et al., 2020), thereby addressing an empirical gap. The process of aging is an inevitable condition that presents a series of challenges for older healthcare professionals. However, those physicians with a proactive personality are able to identify and leverage their strengths in the context of adversity, thereby enhancing their capacity to identify and create opportunities (Chen et al., 2021). According to lifespan development theory, a proactive personality encourages older workers to take initiative in coping with declines in competence and motivation, which may result in successful aging (Bakker et al., 2012). Our findings confirm that a proactive personality positively influences the SAW of aging physicians in hospitals, validating the applicability of lifespan development theory in this context and enriching the understanding of the association between individual factors and SAW.

Secondly, we discovered that job crafting mediates the relationship between proactive personality and SAW, a connection that previous research has not directly established. While prior research has substantiated the assertion that employees with proactive personalities exhibit a greater propensity for engaging in job crafting (Rudolph et al., 2017). Furthermore, job crafting has been demonstrated to have a beneficial impact on career growth (Miao et al., 2023), which is an essential component of SAW. This study further explored the mechanisms by which proactive personality influences SAW, identifying job crafting as a mediating factor in this relationship. Older workers with a proactive personality are more inclined to adjust job content and the work environment to align with organizational needs and achieve their own career goals.

Thirdly, this study identified POS as a boundary condition. We indicated that POS would affect the benefits of proactive personality on SAW via job crafting. Our findings empirically confirm the positive moderating role of POS, offering valuable insights for organizations to better assist employees in achieving SAW.

### 5.2 Practical implication

The study builds on the characteristics of healthcare, such as older physicians having extensive clinical experience and continued development in medical science. Thus, we construct an analytical model of SAW that is applicable to older physicians and provides recommendations for them to achieve successful aging. Our study demonstrates the impact of older physicians' individual factors and POS on SAW, clarifies the role of proactive response strategies for individuals, and highlights the important role of organizations in facilitating the enhancement of older physicians' ability to work and develop sustainably. Firstly, it is essential to identify the personality traits of older physicians within the organization. Those who are proactive can be positively guided and adequately supported to enable them to adopt proactive strategies to adapt to the process of Aging. Secondly, it is crucial to cultivate an environment that encourages job crafting among older physicians, enabling them to achieve a more optimal person-job fit (Zacher and Yang, 2016), and facilitating the late-career development of older physicians. Finally, Organizations should improve older physicians' autonomy and engagement, and maintain a better match with their work (Earl et al., 2018) to enhance their sense of control over their work, contributing to greater job satisfaction and overall wellbeing (Cheung and Wu, 2012).

### 5.3 Limitation and future research

While our findings offer valuable initial empirical evidence, the study also has several limitations. Primarily, data were collected at a single time point, which restricts the ability to draw definitive. To address this limitation, future research should consider adopting a longitudinal design. Secondly, the respondents in this study were physicians aged 50 and above working in hospitals, which may limit the external validity of the findings. Future research could broaden the age range of participants and include professionals from other sectors to enhance generalizability. Thirdly, the use of self-reported measures for variables may have introduced response bias, which could potentially compromise the objectivity and accuracy of our findings. Future studies should incorporate data from external sources, such as supervisors and colleagues, or utilize objective indicators. Finally, this study explored the mechanisms linking proactive personality to SAW. Given the significant direct effect of proactive personality on SAW, future research could investigate how other individual factors influence SAW, thereby deepening our understanding of its underlying mechanisms.

## 6 Conclusion

This study contributes to the existing literature by examining the relationship between proactive personality and SAW among older physicians. The empirical findings indicate that proactive personality is positively associated with SAW, with job crafting serving as a mediator in this relationship. Furthermore, POS is recognized as a moderating factor. The study enhances our understanding of the mechanisms through which personal characteristics influence SAW. It also provides guidance on management practices such as maintaining optimal working conditions and the development of innovative approaches to work for older physicians.

## Data Availability

The original contributions presented in the study are included in the article/supplementary material, further inquiries can be directed to the corresponding author.
